# Supplementary material for: MetaRibo-Seq measures translation in microbiomes
Source: Nat Commun. 2020 Jun 29;11:3268. doi: 10.1038/s41467-020-17081-z (PMC7324362; doi:10.1038/s41467-020-17081-z)
Supplement: Supplementary file 10 — Supplementary Data 7 [file 41467_2020_17081_MOESM10_ESM.zip › File2/Confidence_High_Taxonomy/333727_out.krona.html]

Javascript must be enabled to view this page.

members
magnitude
magnitudeUnassigned
count
unassigned
taxon
rank

333727\_out

23

23
superkingdom
2

1239
phylum
23

186801
23
class

186802
23
order

23
family
186803

23
genus
841

166486
species

SRS013158\_contig\_number\_3351SRS014923\_contig\_number\_46852SRS014948\_contig\_number\_348SRS015065\_contig\_number\_35765SRS015190\_contig\_number\_14575SRS015578\_contig\_number\_27059SRS015579\_contig\_number\_22055SRS015960\_contig\_number\_16040SRS019267\_contig\_number\_788SRS022137\_contig\_number\_5231SRS043701\_contig\_number\_14867SRS054956\_contig\_number\_3875SRS056519\_contig\_number\_18786SRS058070\_contig\_number\_24394SRS076756\_contig\_number\_15944SRS098827\_contig\_number\_contig-100\_945.83952SRS098881\_contig\_number\_contig-100\_297.169771SRS101433\_contig\_number\_3682SRS104485\_contig\_number\_407SRS105153\_contig\_number\_29991SRS142781\_contig\_number\_11324SRS143417\_contig\_number\_13210SRS149879\_contig\_number\_27355
23
